# An active site mutation induces oxygen reactivity in D-arginine dehydrogenase: A case of superoxide diverting protons

**DOI:** 10.1016/j.jbc.2024.107381

**Published:** 2024-05-17

**Authors:** Joanna A. Quaye, Kendall E. Wood, Claire Snelgrove, Daniel Ouedraogo, Giovanni Gadda

**Affiliations:** 1 Department of Chemistry, Georgia State University, Atlanta, Georgia, USA; 2 Biology Department, Morehouse College, Atlanta, Georgia, USA; 3 The Gwinnett School of Mathematics, Science, and Technology, Lawrenceville, Georgia, USA; 4 Department of Biology, Georgia State University, Atlanta, Georgia, USA; 5 Department of the Center for Diagnostics and Therapeutics, Georgia State University, Atlanta, Georgia, USA

**Keywords:** *Pseudomonas aeruginosa* D-arginine dehydrogenase, pH profiles, nonstoichiometric slope, superoxide, superoxide disproportionation, reduced flavin semiquinone, rapid-reaction kinetics, O_2_-driven dehydrogenase activity

## Abstract

Enzymes are potent catalysts that increase biochemical reaction rates by several orders of magnitude. Flavoproteins are a class of enzymes whose classification relies on their ability to react with molecular oxygen (O_2_) during catalysis using ionizable active site residues. *Pseudomonas aeruginosa* D-arginine dehydrogenase (*Pa*DADH) is a flavoprotein that oxidizes D-arginine for *P. aeruginosa* survival and biofilm formation. The crystal structure of *Pa*DADH reveals the interaction of the glutamate 246 (E^246^) side chain with the substrate and at least three other active site residues, establishing a hydrogen bond network in the active site. Additionally, E^246^ likely ionizes to facilitate substrate binding during *Pa*DADH catalysis. This study aimed to investigate how replacing the E^246^ residue with leucine affects *Pa*DADH catalysis and its ability to react with O_2_ using steady-state kinetics coupled with pH profile studies. The data reveal a gain of O_2_ reactivity in the E^246^L variant, resulting in a reduced flavin semiquinone species and superoxide (O_2_^•^**ˉ**) during substrate oxidation. The O_2_^•^**ˉ** reacts with active site protons, resulting in an observed nonstoichiometric slope of 1.5 in the enzyme’s log (*k*_cat_/*K*_m_) pH profile with D-arginine. Adding superoxide dismutase results in an observed correction of the slope to 1.0. This study demonstrates how O_2_^•^**ˉ** can alter the slopes of limbs in the pH profiles of flavin-dependent enzymes and serves as a model for correcting nonstoichiometric slopes in elucidating reaction mechanisms of flavoproteins.

Flavin-dependent enzymes are a class of enzymes that often rely on ionization processes during catalysis (1, 2, 3, 4, 5, 6, 7, 8, 9, 10, 11, 12, 13, 14, 15). Flavoproteins can be classified as oxidases, monooxygenases, and dehydrogenases, depending on their ability to use molecular oxygen (O_2_) as an electron acceptor and the products of their reactions (16, 17). Oxidases use O_2_ as an electron acceptor to produce H_2_O_2_. Monooxygenases insert an oxygen atom from O_2_ into the substrate with H_2_O as a product. Dehydrogenases do not react with O_2,_ or if they do, they reduce O_2_ to superoxide radicals (O_2_^•^**ˉ**) without the production of H_2_O_2_ or H_2_O (18). In flavin-dependent enzymes, the spin-forbidden reaction of the triplet state O_2_ with the singlet state reduced flavin is overcome by successive electron transfers in a step-wise process to yield a caged O_2_^•^**ˉ**/flavin semiquinone radical pair (16, 17, 19, 20, 21). Studies on flavoproteins show that enzymes that react with O_2_, such as oxidases and monooxygenases, overcome the thermodynamically unfavorable generation of the O_2_^•^**ˉ**/flavin semiquinone radical pair by stabilizing the transition state of the O_2_^•^**ˉ**/flavin semiquinone radical pair through electrostatic catalysis using a positive charge close to the flavin C(4a) atom (16, 17, 21, 22, 23, 24).

From structural and mechanistic analysis, a structural motif comprising nonpolar residues in the active site and a positive charge, either in the protein or from the enzyme’s substrate or product, has been identified as a requirement for flavin reactivity with O_2_ to yield the O_2_^•^**ˉ**/flavin semiquinone radical pair (24). In principle, a flavoprotein can gain the ability to react with O_2_, provided these minimum requirements are met. The highly reactive O_2_^•^**ˉ** can then undergo an ionization process to acquire a proton from the enzyme or bulk solvent, yielding a hydroperoxyl radical (HO_2_^•^) with a p*K*_a_ value of 4.8 (25, 26). The key players of ionization processes in several enzymes are solvent water, metal ions, and the side chains of ionizable active site residues (10, 16, 17, 27, 28, 29, 30, 31, 32, 33, 34, 35, 36). For several decades, pH profile and mutagenesis studies have been widely employed to identify and assign p*K*_a_ values to the ionizing groups during enzyme catalysis (37, 38, 39, 40, 41, 42, 43). However, the pH profiles used to identify ionizing groups and assign p*K*_a_ values could be misinterpreted if an enzyme produces highly reactive species such as O_2_^•^**ˉ** that can react with ionized protons during catalysis. Hence, the question remains whether the formation and leakage of O_2_^•^**ˉ** in flavoproteins that react poorly with O_2_ to yield O_2_^•^**ˉ** can affect the pH profiles used in assessing the residues important for enzyme catalysis.

The *Pseudomonas aeruginosa* D-arginine dehydrogenase (*Pa*DADH) is a flavoprotein found in the opportunistic pathogen *P. aeruginosa* (44). *The enzyme* is important for *P. aeruginosa*’s utilization of D-arginine as a primary source of carbon and nitrogen by oxidizing the CN bond between the Cα atom and the amino group of its substrates (45). In recent years, the increased prevalence of multidrug-resistant strains of *P. aeruginosa* has been largely associated with the formation of bacterial biofilms coupled with bacterial antibiotic-resistance mechanisms (46, 47, 48, 49, 50, 51, 52, 53, 54). Studies have identified that increased levels of L-arginine in *P. aeruginosa* reduce bacterial mobility, enhancing the formation of bacterial biofilms required for chronic infections (53, 54, 55, 56, 57, 58, 59, 60, 61, 62, 63, 64). In *P. aeruginosa*, *Pa*DADH exists in a two-enzyme conversion system with L-arginine dehydrogenase, allowing the bacterium to accumulate L-arginine from D-arginine oxidation (65). Thus, *Pa*DADH’s activity influences *P. aeruginosa* to favor the sessile biofilm-forming lifestyle required for chronic infections (63, 64). More recently, the enzyme has emerged as a paradigm for flavin-containing enzymes that oxidize positively charged D-amino acids (66).

*Pa*DADH has broad substrate specificity. The enzyme oxidizes all D-amino acids, except D-aspartate and D-glutamate, to their corresponding α-imino acids, followed by nonenzymatic hydrolysis of the α-imino acid products to yield α-keto acids and ammonia (Fig. 1) (44, 67, 68, 69). *Pa*DADH is a strict dehydrogenase that does not react with O_2_ and follows a ping-pong bi-bi steady-state kinetic mechanism (44, 66). Structural analysis of the enzyme’s active site reveals only polar amino acid residues, including H^48^, Y^53^, E^87^, R^222^, E^246^, Y^249^, and R^305^, and a lack of nonpolar residues (69). The enzyme contains four flexible loops, L1, L2, L3, and L4 (69), with residues Y^53^ of loop L1 and E^246^ of loop L2 involved in a gating interaction that secures the substrate in the active site for catalysis (Fig. 2) (67, 69). Residue E^246^ of loop L2 is located 7.6 Å from the flavin N5 atom, 8.2 Å from the flavin C(4a) atom, and does not participate in flavin reduction (70).

**Figure 1:**
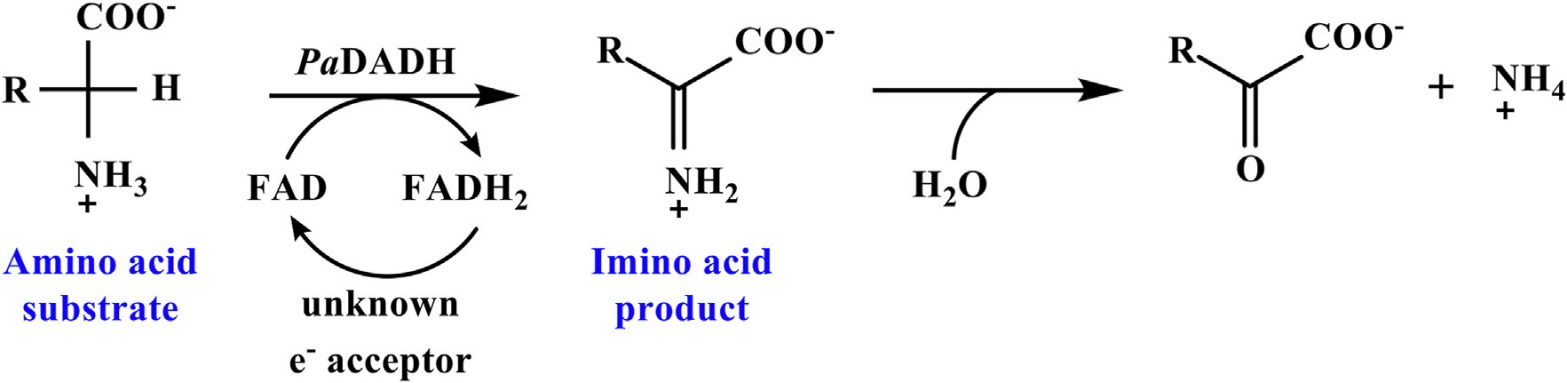
**General reaction scheme of *Pa*DADH.***Pa*DADH, Pseudomonas aeruginosa D-arginine dehydrogenase.

**Figure 2:**
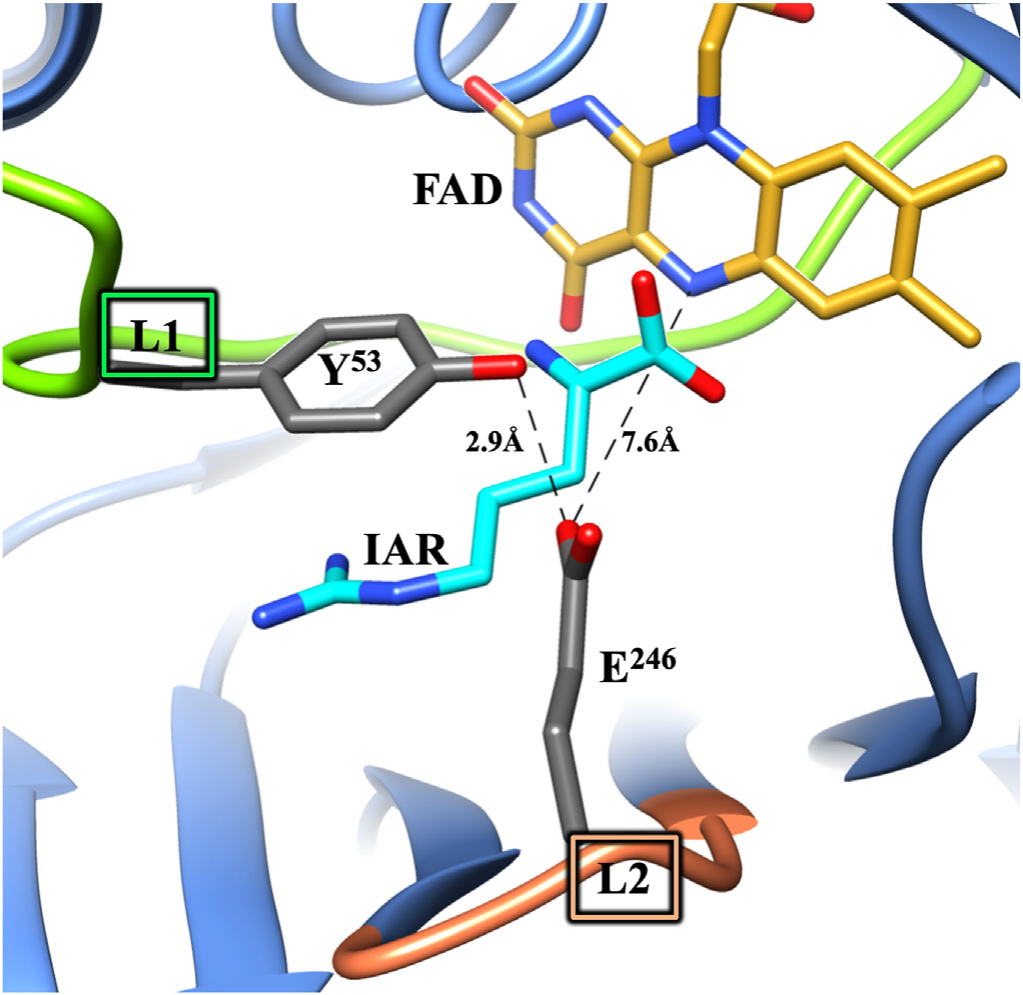
**Gating of *Pa*DADH active site by loop L1 and L2 residues Y**^**53**^**and E**^**246**^**.** Y^53^ and E^246^ are shown in *gray*. All N atoms are shown in *blue*, and all O atoms are in *red*. The FAD cofactor is represented by its isoalloxazine ring with the C atoms in *gold*. IAR represents the iminoarginine product and is shown in *cyan*. The E^246^ residue’s hydrogen bond interaction with Y^53^ and distance from the flavin are shown as *dashed lines*. Loop L2 is shown in *coral*, and loop L1 is shown in *green*. The PDB file 3NYE was visualized and analyzed using the UCSF Chimera software (110). *Pa*DADH, *Pseudomonas aeruginosa* D-arginine dehydrogenase.

In this study, the *Pa*DADH residue E^246^ has been mutated to leucine to yield the glutamate 246 to leucine mutant (E^246^L) variant enzyme. The effects of the E^246^L mutation on the enzyme’s catalysis and ability to react with O_2_ have been investigated using rapid-reaction kinetics, steady-state kinetics, and pH profile studies. Additionally, the mutation’s effects on the slopes of the pH profile plots have been explored.

## Results

### Rapid-reaction kinetics of the *Pa*DADH E^246^L variant enzyme with D-leucine as a substrate

**Figure 3:**
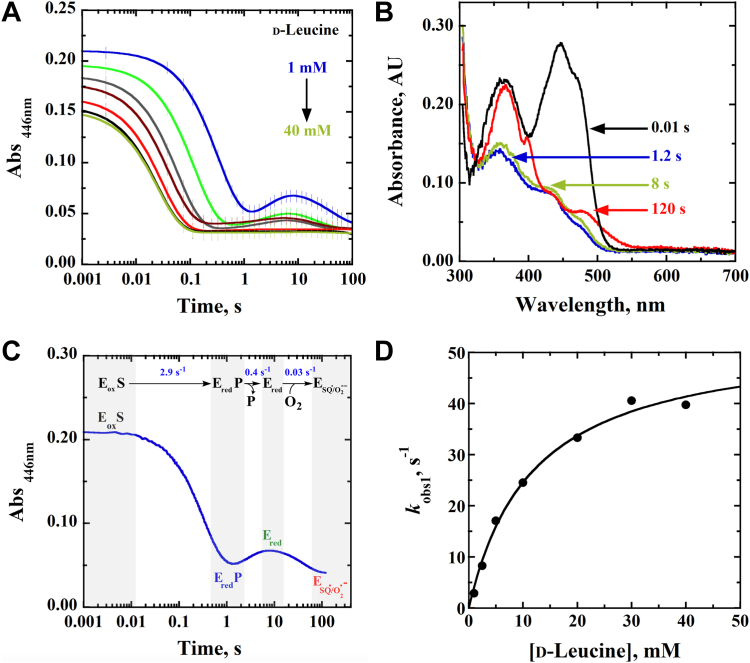
**Aerobic reductive-half reaction of the *Pa*DADH E**^**246**^**L variant enzyme.***A*, stopped-flow traces of the absorbance changes at 446 nm at different D-leucine concentrations (1–25 mM) fit with Equation 1. Each trace is the average of triplicate runs at each substrate concentration. For clarity, one out of every 100 experimental points is shown (*vertical lines*). Note the log time scale. *B*, time-resolved UV-visible absorption spectra of the various flavin species generated during the aerobic reduction of the *Pa*DADH E^246^L variant enzyme with D-leucine. The reaction was monitored over 120 s (s) upon mixing 1 mM D-leucine with the E^246^L variant enzyme of *Pa*DADH in the presence of atmospheric O_2_. The *black spectrum* represents the oxidized enzyme; the *red spectrum* represents the reduced flavin semiquinone; the *green* and the *blue spectra* represent the fully reduced flavin. *C*, time map for the various flavin species generated during the aerobic reductive-half reaction of the *Pa*DADH E^246^L variant enzyme with 1 mM D-leucine. *D*, the observed rate constant for flavin reduction as a function of D-leucine concentration under aerobic conditions fit with Equation 2. The single point shown at each substrate concentration is the *k*_obs_ value obtained from the fit of the average of triplicate runs with Equation 1 yielding an error of ≤5%. The assay was performed in 20 mM NaPP_i_, pH 10.0, using an SF-61DX2 Hi-Tech KinetAsyst high-performance stopped-flow spectrophotometer thermostated at 25 ^o^C and equipped with a photomultiplier detector under aerobic conditions. The instrumental dead time is 2.2 ms. E246L, glutamate 246 to leucine mutant; *Pa*DADH, *Pseudomonas aeruginosa* D-arginine dehydrogenase.

Since ∼80% of the flavin reduction of the *Pa*DADH E^246^L variant enzyme with D-arginine occurs in the mixing time (2.2 ms) of the stopped-flow spectrophotometer (42, 71), the flavin reduction of the enzyme was investigated with D-leucine as the reducing substrate. The time-resolved aerobic reduction of the *Pa*DADH E^246^L variant enzyme by D-leucine was investigated under pseudo-first-order conditions by monitoring the loss of the oxidized flavin’s absorbance at 446 nm. The resulting stopped-flow traces showed three distinct reaction phases at [D-leucine] ≤10 mM. As expected for flavin reduction, the absorbance at 446 nm decreased; however, there was a subsequent transient increase in the 446 nm absorbance (Fig. 3*A*). The stopped-flow traces were fit with triple exponentials (Equation 1).

Analysis of the time-resolved UV-visible absorption spectra of the various flavin species generated during the aerobic reduction of the *Pa*DADH E^246^L variant enzyme with D-leucine showed an oxidized flavin spectrum at 0.01 s with a λ_max_ at 446 nm and a second peak at 367 nm. After 1.2 s, there was an observed quenching of the λ_446 nm_ and λ_367 nm_ peaks to yield a spectrum with a λ_max_ at 367 nm, consistent with a reduced flavin being present. After 8 s, there was an observed slight increase in the reduced flavin absorbance between 367 nm and 500 nm, although the overall spectral characteristics of the reduced flavin at 1.2 s were maintained. After 120 s, there were three observed peaks in the flavin spectrum at 367 nm, 394 nm, and 480 nm, consistent with the formation of a reduced flavin semiquinone species (Fig. 3*B*).

The first phase of the time-resolved aerobic reduction of the *Pa*DADH E^246^L variant with D-leucine, characterized by the bleaching of the oxidized flavin absorption at 446 nm, was assigned to flavin reduction. The next phase, characterized by the transient gain of absorbance at 446 nm, was assigned to the imino acid product release. The last phase, showing a quenching of absorbance at 446 nm, was assigned to the reaction of the reduced flavin with O_2_ (Fig. 3*C*), hinting at the formation of a caged O_2_^•^**ˉ**/flavin semiquinone radical pair during the aerobic reduction of the *Pa*DADH E^246^L variant enzyme. The lack of a transient increase in the 446 nm absorbance at [D-leucine] ≥10 mM can be explained as a likely formation of enzyme–substrate complexes between the reduced enzyme and excess substrate following the imino acid product release, which prevents the reduced flavin from reacting with O_2_.

To obtain the observed rate constant (*k*_obs1_) for flavin reduction, the rate constants for the first phase at any given substrate concentration were fit with Equation 2, yielding a zero y-intercept hyperbolic dependence of the *k*_obs1_ parameter on D-leucine concentration (Fig. 3*D*), allowing for the determination of the limiting rate constant for flavin reduction (*k*_red_) and the apparent equilibrium constant for the dissociation of the substrate from the Michaelis complex (*K*_d_). The resulting kinetic parameters are shown in Table 1. There was no observed dependence of the *k*_obs2_ and *k*_obs3_ parameters on [D-leucine].

**Table 1:** Rapid-reaction kinetic parameters of the *Pa*DADH E^246^L variant enzyme with D-leucine as substrate

Kinetic parameter	aAerobic	bAnaerobic
*k*_red_ (s^−1^)	54 ± 3	35 ± 1
*K*_d_ (mM)	12 ± 2	12 ± 1
*k*_red_/*K*_d_ (M^−1^s^−1^)	4600 ± 400	2900 ± 75

a Reductive-half reaction kinetics were measured at varying concentrations of D-leucine under aerobic conditions. Assays were performed in 20 mM NaPP_i_, pH 10.0, at 25 ^o^C. The kinetic parameters’ values were obtained after fitting the kinetic data with Equation 2.

b Previously reported data for the *Pa*DADH E^246^L variant enzyme under anaerobic conditions (70).

### O_2_ reactivity studies of the *Pa*DADH E^246^L variant enzyme

**Figure 4:**
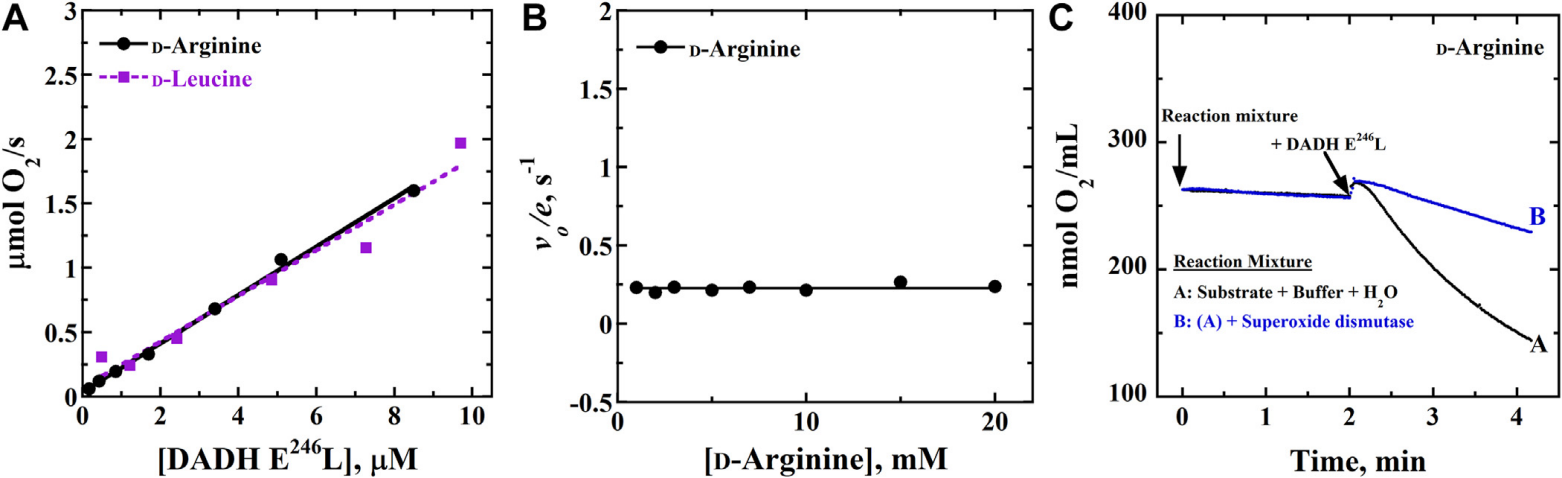
**O**_**2**_**reactivity studies of the *Pa*DADH E**^**246**^**L variant enzyme.***A*, plot of the initial velocity of the *Pa*DADH E^246^L variant enzyme’s reactivity with O_2_ as a function of enzyme concentration at fixed 5 mM D-arginine in *black* and 25 mM D-leucine in *purple*. *B*, the dependence of the rate of O_2_ reactivity of the *Pa*DADH E^246^L variant enzyme as a function of D-arginine concentration. *C*, the effect of superoxide dismutase on the *Pa*DADH E^246^L variant enzyme’s reaction with O_2_ with 5 mM D-arginine as substrate. The *black trace* represents the O_2_ reactivity without superoxide dismutase. The *blue trace* represents the experiment performed in the presence of superoxide dismutase. The assays were carried out in 20 mM NaPP_i_, pH 8.0, using a Clark-type oxygen electrode system, thermostated at 25 ^o^C. E246L, glutamate 246 to leucine mutant; *Pa*DADH, *Pseudomonas aeruginosa* D-arginine dehydrogenase.

The ability of the *Pa*DADH E^246^L variant enzyme to react with O_2_ as an electron acceptor was explored by measuring the initial velocities of the enzymatic reaction at various enzyme concentrations with 25 mM D-leucine as substrate and O_2_ as the electron acceptor. The data yielded a positive-sloped linear dependence of the initial velocities of enzyme activity as a function of enzyme concentration. Similar results were obtained when the assay was repeated with D-arginine as substrate (Fig. 4*A*).

A separate experiment to investigate the dependence of the O_2_ reactivity on D-arginine concentration yielded a flat line with a rate constant for the oxygen-driven dehydrogenase activity† of 0.23 s^−1^, irrespective of the substrate concentration tested (Fig. 4*B*). When superoxide dismutase was introduced into the reaction assay to determine the O_2_ species generated during the enzyme’s turnover with O_2_ as the electron acceptor, there was an observed decrease in the initial velocity of the enzymatic reaction with D-arginine (Fig. 4*C*). Similar results were obtained when the above assays were repeated with D-leucine as substrate (data not shown).

**Figure 5:**
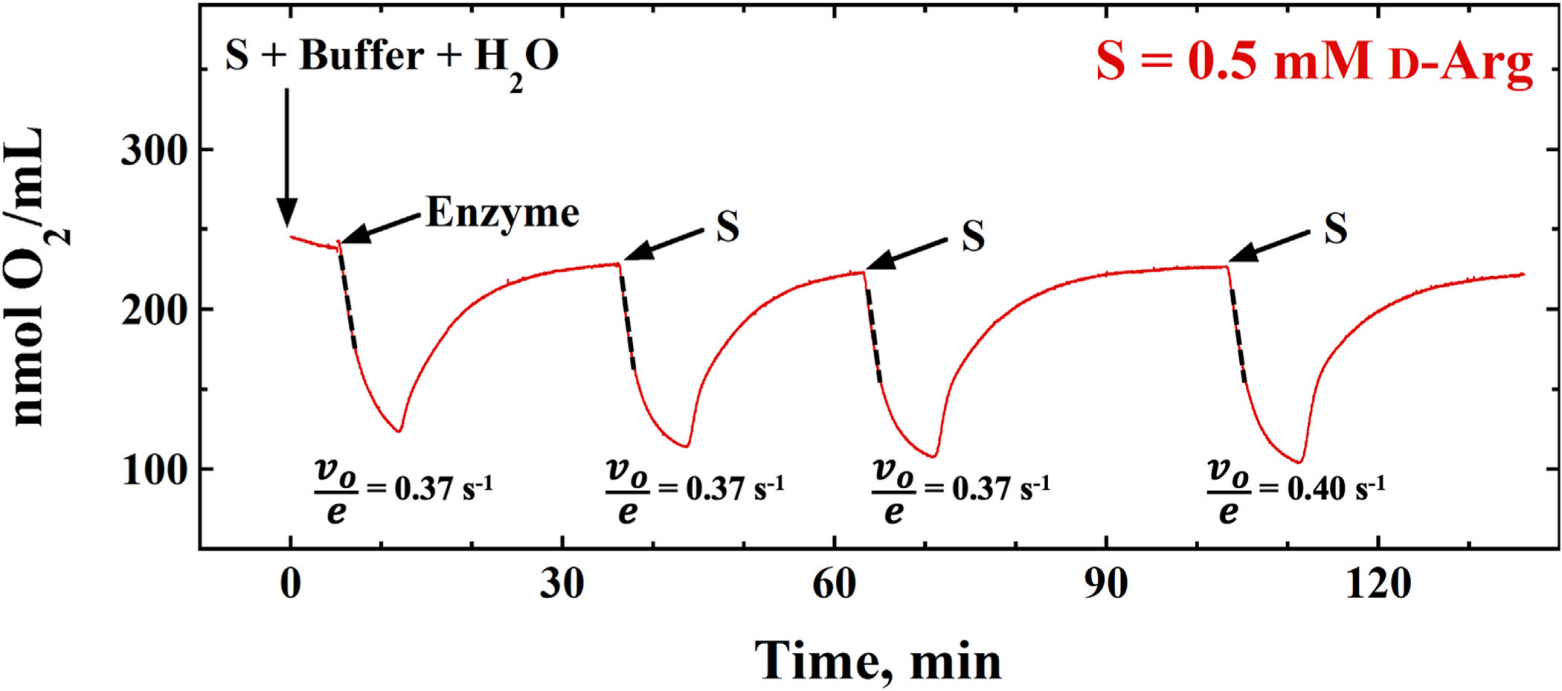
**Effect of the *Pa*DADH E**^**246**^**L-generated O**_**2**_^**•**^**ˉ on the turnover ability of the enzyme.** O_2_ consumption and regeneration cycles were carried out with fixed 6 μM enzyme and 0.5 mM d-arginine. The steady-state regions are shown in *black dotted lines* and the initial rates of D-arginine oxidation for each turnover cycle is shown below as *v*_*o*_*/e*. The assay was performed in 20 mM NaPP_i_, pH 8.0, with O_2_ as the electron acceptor, using a Clark-type oxygen electrode system at 25 ^o^C. E246L, glutamate 246 to leucine mutant; *Pa*DADH, *Pseudomonas aeruginosa* D-arginine dehydrogenase.

The steady-state kinetic studies of the *Pa*DADH E246L variant enzyme reported in a previous study yielded a *k*_cat_ value of 265 ± 5 s^−1^, a *k*_cat_/*K*_m_ value of 871,000 ± 35,000 M^−1^s^−1^, and a *K*_m_ value of 0.30 ± 0.02 mM with D-arginine (70). Hence, to determine the effect of the enzyme-generated O_2_^•^**ˉ** on *Pa*DADH E^246^L turnover with D-arginine, assays of the variant enzyme with O_2_ as electron acceptor and 0.5 mM D-arginine as substrate were carried out under steady-state conditions. The *Pa*DADH E^246^L variant underwent multiple turnover cycles spanning 2 h upon reintroduction of D-arginine into the reaction mixture (Fig. 5). For all enzyme turnover cycles, the initial rate of the oxygen-driven dehydrogenase activity was ∼0.4 s^−1^. Similar rates for D-arginine oxidation were observed when the experiment was repeated using 0.15 and 0.24 mM D-arginine (data not shown).

**Figure 6:**
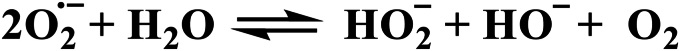
**The disproportionationof O2•ˉ in water.**

Additionally, O_2_ regeneration was observed in every enzyme turnover cycle before the D-arginine substrate was reintroduced (Fig. 5). The observed oxygen regeneration can be explained by a proton-dependent O_2_^•^**ˉ** disproportionation to yield hydroperoxide anion HO_2_**ˉ**, hydroxide HO**ˉ**, and O_2_ (Fig. 6) (72), followed by a slow re-equilibration of the solution in the O_2_ electrode chamber toward atmospheric oxygen. The observed nonzero O_2_ level after enzyme turnover with D-arginine can be explained by the O_2_^•^**ˉ** disproportionation reaction leading to O_2_ accumulation that gradually overturns the O_2_ consumption of the D-arginine oxidation.

### Effects of pH on the *k*_cat_/*K*_m_ parameter of *Pa*DADH E^246^L

**Figure 7:**
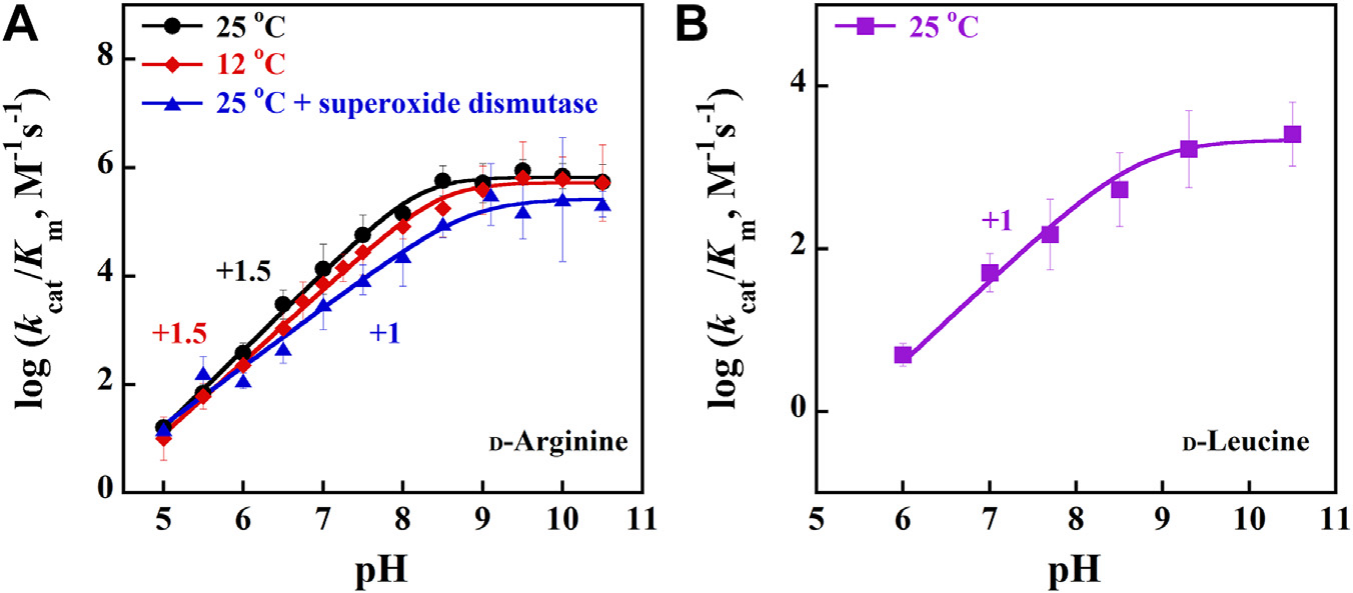
**Effects of superoxide dismutase and pH on the *k***_**cat**_**/*K***_**m**_**parameter of the *Pa*DADH E**^**246**^**L variant with D-arginine or D-leucine as substrate.***A*, pH dependence of the *k*_cat_/*K*_m_ parameter with D-arginine. *B*, pH dependence of the *k*_cat_/*K*_m_ parameter with D-leucine. Activity assays were carried out with varying concentrations of D-arginine or D-leucine as a substrate and fixed PMS as an artificial electron acceptor at 1 mM from pH 5.0 to 10.5 in 20 mM NaPP_i_. Assays without superoxide dismutase are shown in *black* and *red* for D-arginine at 25 ^o^C and 12 ^o^C, respectively, and *purple* for D-leucine at 25 ^o^C. The D-arginine assay with superoxide dismutase at 25 ^o^C is shown in *blue*. The D-arginine plots were obtained by fitting the kinetic data to Equation 3 for the *k*_cat_/*K*_m_ parameter without superoxide dismutase at 12 ^o^C and 25 ^o^C, and Equation 4 for the *k*_cat_/*K*_m_ parameter with superoxide dismutase. The D-leucine plot was obtained by fitting the kinetic data with Equation 4. The observed p*K*_a_ values for the various assays are recorded in Table 2 for D-arginine and Table 3 for D-leucine. *Pa*DADH, *Pseudomonas aeruginosa* D-arginine dehydrogenase; PMS, phenazine methosulfate.

Due to the O_2_ reactivity and formation of the flavin semiquinone species at [D-leucine] ≤10 mM during the aerobic reduction of the *Pa*DADH E^246^L variant, the kinetic investigations for the steady-state pH effects focused only on the *k*_cat_/*K*_m_ parameter, which reports on the enzyme’s behavior at low substrate concentrations and probes the free enzyme. Thus, to understand the effects of pH on *Pa*DADH E^246^L’s substrate capture, the steady-state reactions of the enzyme were investigated with D-arginine or D-leucine as the substrate from pH 5.0 to 10.5, with phenazine methosulfate (PMS) as an artificial electron acceptor since the physiological electron acceptor for *Pa*DADH activity is not known. The plots of the log values of the *k*_cat_/*K*_m_ parameter showed an increase in the *k*_cat_/*K*_m_ parameter with increasing pH, a pH-independent region above pH 9.0, and an observed p*K*_a_ value for a basic group between 8.2 and 8.8 (Fig. 7 and Table 2).

**Table 2:** Effects of superoxide dismutase on the pH effects on the steady-state kinetic parameters of the *Pa*DADH E^246^L variant enzyme with D-arginine as substrate^a^

*k*_cat_/*K*_m_				
Condition	C_H_	p*K*_a_	Slope	R^2^
25 ^o^C	665,000 ± 78,000	8.2 ± 0.1	1.5 ± 0.1	0.997
25 ^o^C + superoxide dismutase	263,000 ± 80,000	8.8 ± 0.2	1.1 ± 0.1	0.983
12 ^o^C	531,000 ± 64,000	8.5 ± 0.1	1.4 ± 0.1	0.997

a Enzymatic activities were measured at varying concentrations of D-arginine and fixed 1 mM PMS. Reactions were carried out in 20 mM sodium pyrophosphate. The *k*_cat_/*K*_m_ parameter values were obtained after fitting the kinetic data with Equation 3.

For the D-arginine substrate, the *k*_cat_/*K*_m_ pH profile at 25 ^o^C fit with Equation 3 yielded a plot with a nonstoichiometric slope of +1.5 for the increasing limb, with an observed p*K*_a_ value for a basic group of ∼8.2. When the assay was repeated at 12 ^o^C to investigate the effect of temperature on the slope of the *k*_cat_/*K*_m_ pH profile plot, similar results were obtained, suggesting that temperature does not contribute to the observed slope. When the effect of the enzyme-generated O_2_^•^**ˉ** on the slope of the *k*_cat_/*K*_m_ pH profile plot was investigated by adding 200 to 500 units of superoxide dismutase to each apparent steady-state reaction mixture at 25 ^o^C, the *k*_cat_/*K*_m_ pH profile plot fit with Equation 3 yielded a stoichiometric value of +1 (Table 2).

The effect of pH on the *k*_cat_/*K*_m_ pH profile of *Pa*DADH E^246^L with D-leucine at 25 ^o^C fit with Equation 4 yielded a kinetic plot with a slope of +1 for the increasing limb, with an observed p*K*_a_ value for a basic group of ∼8.7 (Table 3) and a pH-independent region above pH 9.0 (Fig. 7).

**Table 3:** Effects of pH on the steady-state kinetic parameters of the *Pa*DADH E^246^L variant enzyme with D-leucine as substrate at 25 ^o^C^a^

Kinetic parameter	C_H_	p*K*_a_	R^2^
*k*_cat_/*K*_m_	2200 ± 500	8.7 ± 0.1	0.987

a Enzymatic activities were measured at varying concentrations of D-leucine and fixed 1 mM PMS. Reactions were carried out in 20 mM sodium pyrophosphate. The *k*_cat_/*K*_m_ parameter values were obtained after fitting the kinetic data with Equation 4.

## Discussion

This study aimed to investigate the effects of the E^246^L mutation on the ability of *Pa*DADH to react with O_2_. The study also investigated the effect of the E^246^L mutation on the catalysis of *Pa*DADH using steady-state kinetics coupled with pH profile studies. The data demonstrate that upon the E^246^L mutation, *Pa*DADH, which is a strict dehydrogenase that does not react with O_2_, gains the ability to react with O_2_, although poorly, to yield a reduced flavin semiquinone and O_2_^•^**ˉ** (66, 67). Consequently, the *Pa*DADH E^246^L variant turns over with O_2_ as an electron acceptor through an alternative dehydrogenase pathway. Following the acquired O_2_ reactivity, the variant enzyme yields a nonstoichiometric slope in the plot of the log (*k*_cat_/*K*_m_) parameter as a function of pH with D-arginine as substrate. Details on the gain of function and the implications on *Pa*DADH catalysis are discussed below.

The *Pa*DADH E^246^L variant enzyme reacts with O_2_ to form a flavin semiquinone during substrate oxidation. Evidence supporting this conclusion comes from the enzyme-monitored aerobic flavin reduction with D-leucine and oxygen (Fig. 3), showing the formation and decay of an enzyme intermediate between 1.2 and 120 s. Analysis of the spectral data of the flavin reduction revealed a flavin spectrum with a peak at 367 nm, characteristic of a flavin semiquinone species at 120 s (Fig. 3) (73, 74, 75). By comparison, this feature is not observed with the *Pa*DADH wildtype enzyme under both aerobic and anaerobic conditions (42, 44, 66, 67, 69, 76, 77). In the same way, the flavin semiquinone species was not observed with the E^246^L variant enzyme under anaerobic conditions, although similar kinetic parameters as reported for the aerobic flavin reduction in this study were observed: *k*_red_ = ∼50 s^−1^ and *K*_d_ = 12 mM (Table 1) (70). The data are consistent with the *Pa*DADH E^246^L variant reacting with O_2_ to yield the flavin semiquinone after flavin reduction and product release, as evidenced by the observed transient increase in the λ_466 nm_ absorbance of the reduced flavin at 8 s (Fig. 3). The presence or absence of the product in the active site alters the flavin oscillator strength, yielding different absorbance intensities of the flavin 446 nm peak upon product release (78). The observation that the flavin semiquinone was not formed at [D-leucine] ≥10 mM can be explained as a likely binding of the reduced enzyme to excess substrate. At [D-leucine] ≥10 mM, the reduced enzyme likely forms a complex with free unreacted substrate in the bulk solvent to yield the E_red_S complex. Such a complex renders the reduced flavin unavailable, preventing the free reduced flavin from reacting with O_2_ to yield the semiquinone.

The reactivity of the *Pa*DADH E^246^L variant enzyme with O_2_ yields O_2_^•^ˉ during catalysis. Evidence supporting this conclusion comes from the rapid-reaction kinetics with D-leucine and the steady-state enzymatic assay of the *Pa*DADH E^246^L variant enzyme with O_2_ as the electron acceptor with D-arginine or D-leucine as substrate, with and without superoxide dismutase (Figs. 3 and 4). The plot of the initial velocity of the E^246^L variant enzyme as a function of time showed a decrease in O_2_ consumption in the presence of superoxide dismutase (Fig. 4), consistent with a superoxide dismutase–mediated conversion of the *Pa*DADH E^246^L-generated O_2_^•^**ˉ** to O_2_, which overturns O_2_ consumption (25, 72, 75, 79, 80, 81, 82, 83, 84, 85, 86, 87). The formation of O_2_^•^**ˉ** is concomitant with the observed stabilization of a flavin semiquinone species (*vide supra*) during the aerobic reduction of *Pa*DADH E^246^L to yield the O_2_^•^**ˉ**/flavin semiquinone radical pair. The O_2_^•^**ˉ**/flavin semiquinone radical pair likely arises from a one-electron reduction of O_2_ by the reduced flavin required to overcome the spin-forbidden reaction of the triplet state O_2_ with the singlet state reduced flavin during *Pa*DADH E^246^L reactivity with O_2_, as reported for flavin-dependent enzymes (16, 17, 19, 20, 21, 75, 88).

**Figure 8:**
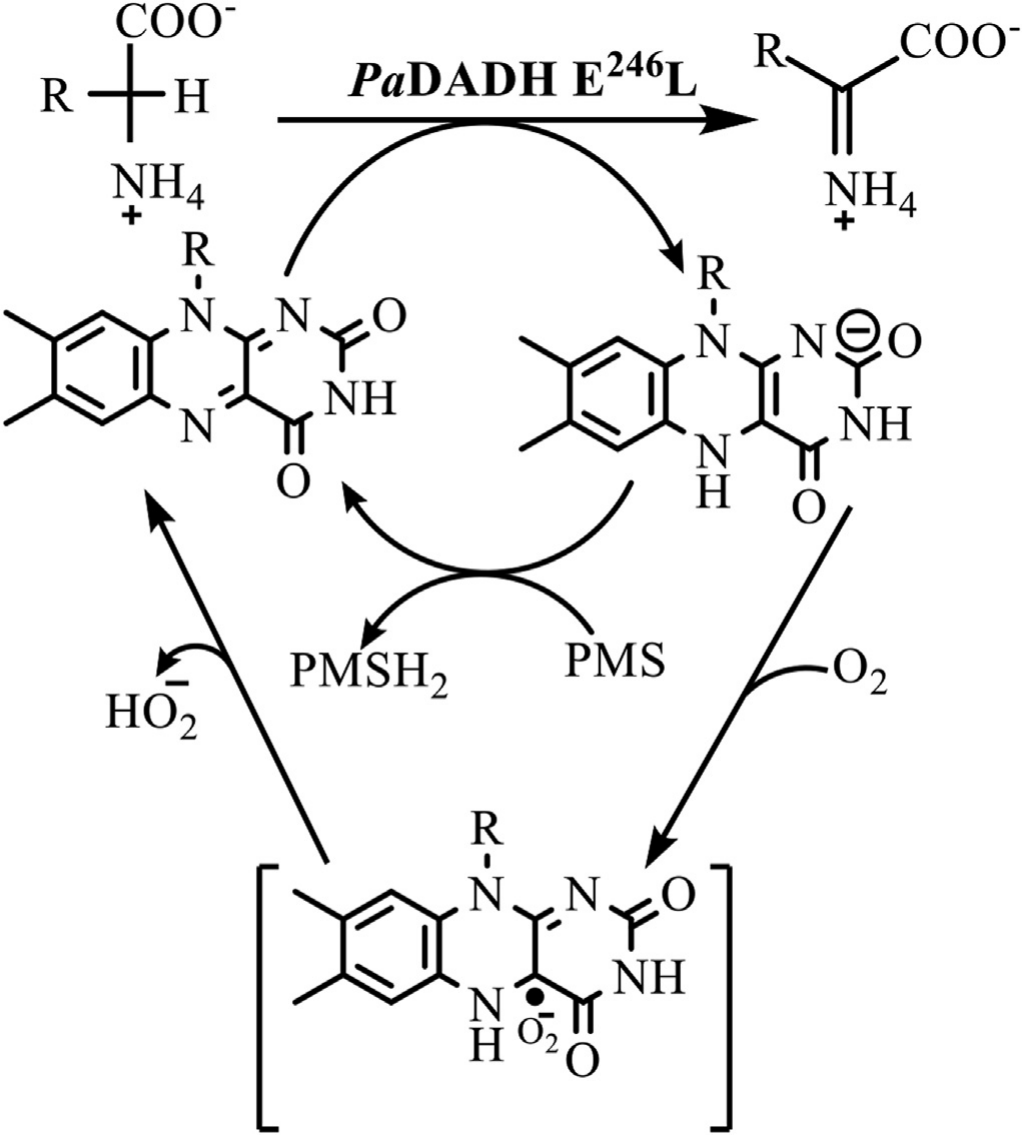
**Proposed reaction scheme of the *Pa*DADH E246L dehydrogenase activity with PMS or O2 as an electron acceptor during turnover.** A hydride transfer from the amino acid substrate reduces the enzyme-bound oxidized flavin to yield the reduced flavin and the imino acid product. The reduced flavin is then re-oxidized through a PMS-driven or an O2-driven dehydrogenase activity. For the PMS-driven turnover, the reduced flavin is re-oxidized by PMS to yield PMSH2, restoring the enzyme to its resting state. For the O2-driven turnover, the reactivity of O2 with the reduced flavin yields the highly reactive caged O2•ˉ/flavin semiquinone radical pair. The flavin semiquinone then donates a hydrogen atom to the O2•ˉ, yielding HO2ˉ and the re-oxidized flavin in the resting state of the enzyme. E246L, glutamate 246 to leucine mutant; O2•ˉ, superoxide radicals; PMS, phenazine methosulfate.

The *Pa*DADH E^246^L variant enzyme undergoes multiple turnover cycles with O_2_. This conclusion is supported by the steady-state assays of *Pa*DADH E^246^L with D-arginine or D-leucine as substrate and O_2_ as electron acceptor. The positive-sloped linear dependence of the plot of the enzymatic initial velocities against enzyme concentration (Fig. 4) demonstrates that *Pa*DADH E^246^L turns over with O_2_. Accordingly, we propose that *Pa*DADH E^246^L follows an O_2_-driven dehydrogenase mechanism (Fig. 8). In the O_2_-driven dehydrogenase mechanism, a hydride transfer from the amino acid substrate reduces the enzyme-bound oxidized flavin to yield the reduced flavin and the imino acid product. The reduced flavin then reacts with O_2_ after product release to form the caged O_2_^•^**ˉ**/flavin semiquinone radical pair. The flavin semiquinone subsequently donates a hydrogen atom to O_2_^•^**ˉ**, producing HO_2_**ˉ** and the re-oxidized flavin. The observation that the initial rates of *Pa*DADH E^246^L turnover with D-arginine and O_2_ remained unchanged at ∼0.4 s^−1^ for 2 h (Fig. 5) suggests that the *Pa*DADH E^246^L-generated O_2_^•^**ˉ** does not compromise the enzyme’s ability to turnover. The sustained catalytic integrity of *Pa*DADH E^246^L can then be explained as a likely prevention of the accumulation of the highly reactive O_2_^•^**ˉ** that may damage the enzyme. A likely mechanism preventing O_2_^•^**ˉ** accumulation is the disproportionation of O_2_^•^**ˉ**, which yields HO_2_**ˉ**, HO**ˉ**, and O_2_ (Fig. 6) (*vide supra*). Moreover, the conversion of O_2_^•^**ˉ** to HO_2_**ˉ** following a hydrogen transfer from the flavin semiquinone during the O_2_-driven dehydrogenase mechanism cannot be ruled out.

The O_2_-driven dehydrogenase activity of *Pa*DADH E^246^L with a rate of 0.23 s^−1^, irrespective of the substrate and concentration tested, can be explained as a likely saturation of the *Pa*DADH E^246^L variant with the amino acid substrate during turnover with O_2_ as the electron acceptor. The data agree well with a recent report investigating the role of the E^246^ residue in *Pa*DADH catalysis in which a small O_2_-driven dehydrogenase activity of ∼0.2 s^−1^ was reported for the E^246^L, E^246^G, and E^246^Q variant enzymes (70). In the same study, the kinetic parameters were investigated using PMS as an artificial electron acceptor for *Pa*DADH since the physiological electron acceptor is unknown. The study reported a *k*_cat_ value of ∼270 s^−1^ with PMS and a maximum rate of ∼0.2 s^−1^ with O_2_ for the E^246^L variant enzyme with D-arginine (70). In this way, the previous study demonstrates that the *Pa*DADH E^246^L variant enzyme turns over primarily through a PMS-driven dehydrogenase mechanism, not an O_2_-driven dehydrogenase mechanism (Fig. 8). Hence, the O_2_-driven dehydrogenase activity does not affect the PMS-driven dehydrogenase activity of *Pa*DADH E^246^L.

The E^246^L variant-generated O_2_^•^ˉ diverts protons from the active site during D-arginine oxidation. This conclusion is supported by the steady-state pH profiles of the *Pa*DADH E^246^L variant with D-leucine and D-arginine in the presence and absence of superoxide dismutase (Fig. 7). The pH profile of the *k*_cat_/*K*_m_ parameter with D-leucine yielded a plot with a +1 slope for the increasing limb from low to high pH. Conversely, with D-arginine, the *k*_cat_/*K*_m_ pH profile yielded a nonstoichiometric slope of +1.5 that was corrected to +1 after superoxide dismutase addition. Conventionally, the slope of a kinetic parameter’s pH profile has been used as an indicator for the number of ionizable processes required to complete the catalytic processes probed by the kinetic parameter (10, 11, 12, 13, 14, 89, 90, 91, 92, 93, 94, 95, 96, 97, 98, 99, 100, 101, 102). Thus, the observed differences in the *Pa*DADH E^246^L *k*_cat_/*K*_m_ pH profile slope with D-arginine and D-leucine can be explained in light of different ionization processes being essential for the catalysis of the two substrates.

**Figure 9:**
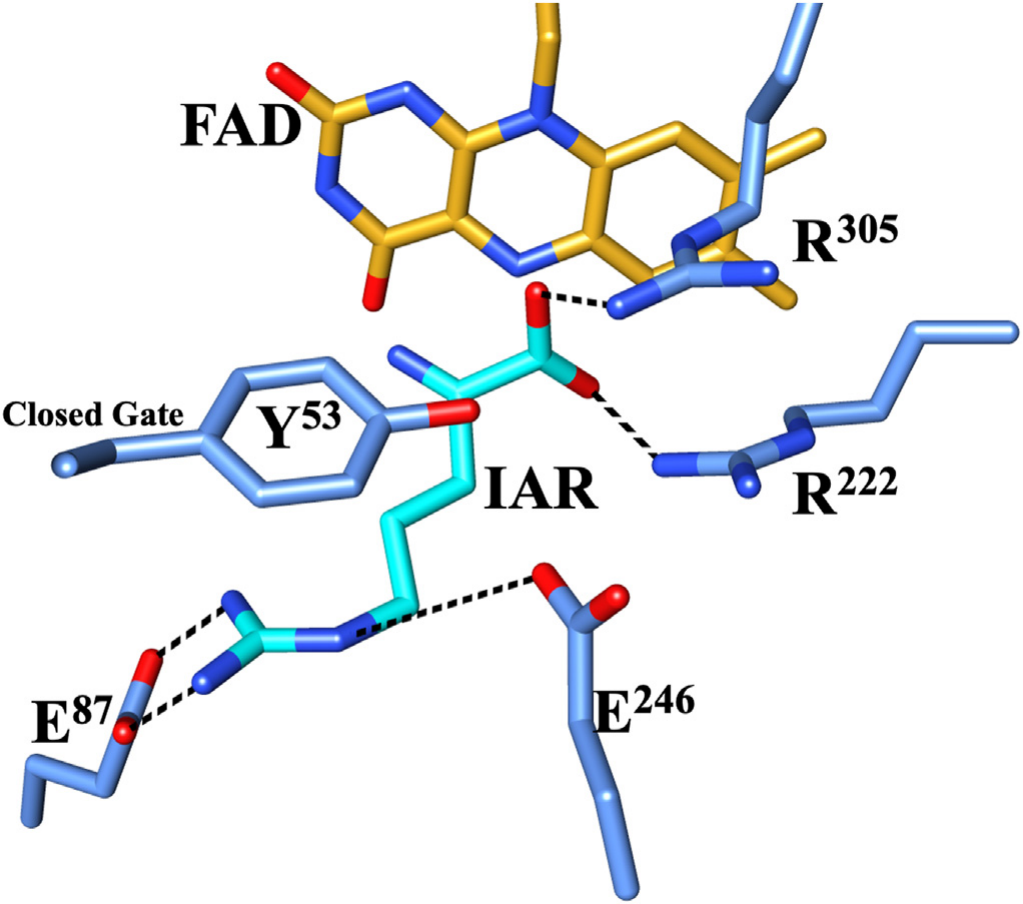
**The active site topology of *Pa*DADH showing substrate interactions and the highly polar active site pocket.** The PDB file 3NYE was visualized and analyzed using the UCSF Chimera software (110). IAR, iminoarginine product; *Pa*DADH, *Pseudomonas aeruginosa* D-arginine dehydrogenase.

With D-arginine, the slope of +1 for the log (*k*_cat_/*K*_m_) pH profile is assigned to the ionization of the substrate’s α-NH_3_^+^ during enzyme catalysis. The observed slope of +1 compared to the slope of +2 previously reported for the wildtype enzyme suggests that the unprotonated E^246^ residue is important for binding D-arginine. However, in a previous study investigating the role of residue E^87^ in *Pa*DADH, the observation of only a single ionizable group with a slope of +1 in the *Pa*DADH E^87^L variant led to the assignment of residue E^87^ as one of the two unprotonated groups required for D-arginine binding, with the substrate’s α-NH_3_^+^ group being the other (77). Altogether, the data portray the requirement for three groups: the substrate’s α-NH_3_^+^, E^87^, and E^246^ for the *k*_cat_/*K*_m_ kinetic parameter during D-arginine oxidation. With the substrate being the commonality between all enzyme forms under discussion: wildtype, E^87^ variant, and E^246^ variant, the first ionizable group can be unequivocally assigned to the substrate’s α-NH_3_^+^ group. Thus, given that the wildtype enzyme reports only two ionizable groups for the *k*_cat_/*K*_m_ pH profile, the assignment of the second group must be critically assessed. From the *Pa*DADH crystal structure (Fig. 9), both E^87^ and E^246^ interact with the guanidinium side chain and are important for D-arginine binding and imino arginine release (70, 77). Since the guanidinium charge is delocalized, we propose that the second ionizable group reflects not one but the joint ionization of the E^87^ and E^246^ residues to ensure maximal binding of D-arginine in the wildtype enzyme. Thus, mutation of either E^87^ or E^246^ yields similar and nonadditive effects on the log (*k*_cat_/*K*_m_) pH profile as reported for His^256^ and Asp^266^ of the carbohydrate-binding domain in rat hepatic lectin-1 (103).

**Figure 10:**
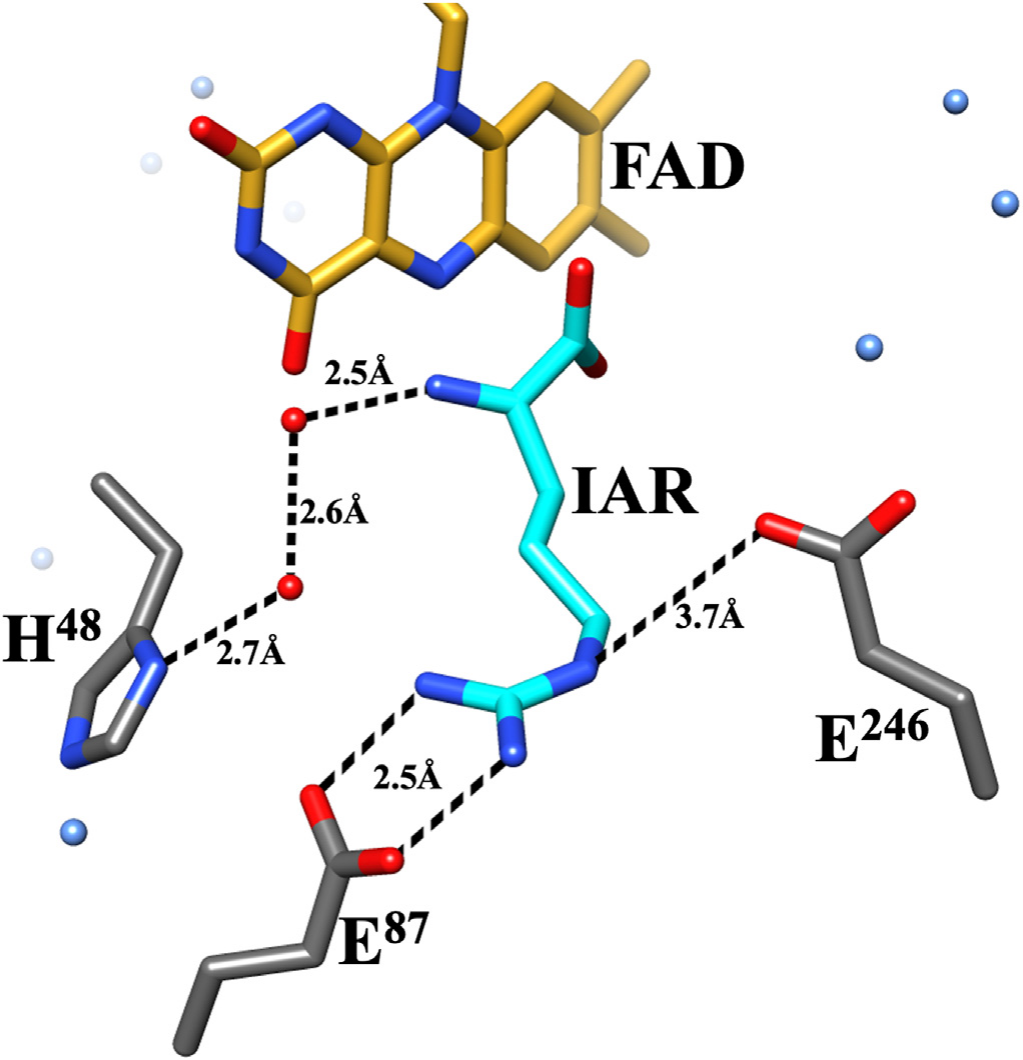
**The proton-relay network of *Pa*DADH.** The PDB file 3NYE was visualized and analyzed using the UCSF Chimera software (110). IAR, iminoarginine product; *Pa*DADH, *Pseudomonas aeruginosa* D-arginine dehydrogenase.

The correction of the log (*k*_cat_/*K*_m_) pH profile’s slope from +1.5 to +1 following superoxide dismutase addition suggests that the O_2_^•^**ˉ** produced by *Pa*DADH E^246^L’s reaction with D-arginine and O_2_ is responsible for the observed nonstoichiometric slope. As the enzyme turns over with D-arginine, the highly reactive O_2_^•^**ˉ** generated during flavin reduction progressively accumulates in the solution due to multiple enzyme turnovers. However, during *Pa*DADH E^246^L oxidation of D-arginine, the proton released from the α-NH_3_^+^ ionization is unavailable for O_2_^•^**ˉ** reactivity due to the H^48^-mediated proton relay network to the bulk solvent (Fig. 10), as previously described for *p-*hydroxybenzoate hydroxylase (66, 77, 104). Thus, the accumulated and highly reactive O_2_^•^**ˉ** most likely reacts with the E^87^ proton to yield HO_2_^•^ during *Pa*DADH E^246^L turnover with low concentrations of D-arginine, thereby favoring E^87^ ionization. The observed steeper and nonstoichiometric slope of +1.5 (Fig. 7) in the log (*k*_cat_/*K*_m_) pH profile, therefore, likely reflects inflated values for the *k*_cat_/*K*_m_ parameter, which probes the free forms of enzymes at low substrate concentrations rather than enzyme–substrate complexes. Due to the O_2_^•^ˉ diversion of the E^87^ ionized protons, there is a partial detection of the E^87^ ionization in the log (*k*_cat_/*K*_m_) pH profile with D-arginine despite the absence of E^246^ (103).

In contrast, the D-leucine substrate has a short and nonpolar side chain and does not require ionization of the E^87^ residue for binding. Thus, there is no observed effect of O_2_^•^**ˉ** on the log (*k*_cat_/*K*_m_) pH profile plot. The observed slope of +1 with an intrinsic p*K*_a_ value of ∼8.5 and common to both D-leucine and D-arginine corresponds to the ionization of the substrate’s α-NH_3_^+^ group that initiates hydride transfer for amine oxidation (42, 77).

The acquired O_2_ reactivity of the E^246^L variant yielding a caged O_2_^•^**ˉ**/flavin semiquinone radical pair can be explained by the modified active site topology of the E^246^L variant. Studies on flavoproteins demonstrate that for an enzyme to react with O_2_, there must be a nonpolar residue and a positive charge close to the flavin cofactor to favor the electrostatics required for O_2_ reactivity (18, 20, 21, 81, 83). In *Pa*DADH, the interaction between the R^222^/R^305^ network and the α-carboxylate group renders the amino acid substrate or imino acid product with a net positive charge in the ligand-bound state (Fig. 9). Nonetheless, in the free form of the enzyme, which is the form that reacts with O_2_, the positive charge likely arises from either R^222^ or R^305^. However, the wildtype enzyme contains only polar amino acid residues, negating one requirement for O_2_ reactivity (66). Conversely, the presence of the nonpolar L^246^ in *Pa*DADH E^246^L provides a suitable active site topology for O_2_ reactivity, having a positive charge and a nonpolar residue (24). Thus, the E^246^L variant enzyme gains the ability to react with O_2_. Similarly, *Pa*DADH reactivity with O_2_ has been recently reported for the Y^249^F variant enzyme after replacing the polar tyrosine 249 residue with the nonpolar phenylalanine residue in the active site (105, 106). However, unlike this study, the Y^249^F variant’s O_2_ reactivity yielded either a flavin N^5^ adduct or a green 6-OH-FAD species. Nonetheless, these studies exemplify how substrates and protein residues dictate the versatility of flavin reactivity to favor specific reactions (105, 106, 107, 108).

In conclusion, this study has used steady-state and rapid-reaction kinetic approaches to investigate the effects of the E^246^L mutation on the ability of *Pa*DADH to react with O_2_ and the role of the generated O_2_^•^**ˉ** in the catalysis of the *Pa*DADH E^246^L variant enzyme. The study demonstrates a mutation-induced gain of *Pa*DADH reactivity with O_2_. pH studies on the steady-state kinetic parameters of the variant enzyme demonstrate that the O_2_^•^**ˉ** generated during *Pa*DADH E^246^L catalysis diverts protons from the active site during D-arginine binding, leading to an observed nonstoichiometric slope of +1.5 in the log (*k*_cat_/*K*_m_) pH profile plot. Conventionally, pH profile slopes of enzyme variants have been the paradigm for identifying ionizable residues involved in enzyme catalysis (37, 38, 39, 40, 41, 42, 43). The technique, which is widely used across the field of enzymology, relies on the identification of integer slopes in pH profile plots for the assignment of catalytic groups after site-directed mutagenesis of enzyme residues. To date, not much information exists on interpreting data from systems with nonstoichiometric pH profile slopes, thereby limiting the applications of pH profiles when elucidating enzyme catalytic mechanisms in such systems. This study provides useful information on correcting and interpreting nonstoichiometric slopes in the pH plots of enzymes that react with O_2_ to form O_2_^•^**ˉ** during catalysis (99, 101, 109). Additionally, this study demonstrates that a charge and nonpolar residue near the flavin can induce oxygen reactivity in an enzyme.

## Experimental procedures

### Materials

*Escherichia coli* strain Rosetta(DE3)pLysS was purchased from Novagen. The QIAprep Spin Miniprep Kit and the QIAquick Polymerase Chain Reaction Purification Kit were obtained from Qiagen. *Pfu* DNA polymerase was purchased from Stratagene, and *Dpn*I was obtained from New England BioLabs. Oligonucleotides were purchased from Sigma Genosys for site-directed mutagenesis and sequencing of the variant genes. Bovine liver superoxide dismutase and PMS were from MilliporeSigma. D-Amino acids were obtained from Alfa-Aesar. All other reagents used were obtained at the highest purity commercially available.

### Site-directed mutagenesis, protein expression, and purification

The E^246^L variant gene of *Pa*DADH was engineered by mutagenic polymerase chain reaction (PCR) with the pET20b(+)/PA3863 plasmid harboring the wildtype gene (*dau*A) as a template. A concentration of 5% dimethyl sulfoxide was added to the PCR reaction mixture to ensure proper separation of the high GC-rich, double-stranded DNA template. Site-directed mutagenesis amplicons were purified using the QIAquick PCR Purification Kit following the manufacturer’s protocol. The purified plasmid was then subjected to endonuclease activity using *Dpn*I at 37 ^o^C for 2 h. The resulting plasmid was used to transform the DH5α strain of *E. coli* cells. The success of the mutation was confirmed by sequencing the gene using the services of Humanizing Genomics Microgen USA Corp in Maryland. The E^246^L variant enzyme of *Pa*DADH was then expressed in *E. coli* Rosetta(DE3)pLysS and purified to homogeneity as previously described for the *Pa*DADH wildtype enzyme in the presence of 10% (v/v) glycerol for enzyme stability and to prevent the loss of the bound FAD cofactor (44). The purified enzyme was stored at −20 ^o^C in 20 mM Tris-Cl, pH 8.0, and 10% glycerol and was found to be active for at least 6 months.

### Rapid-reaction kinetics of the *Pa*DADH E^246^L variant enzyme

To establish the effect of the E^246^L mutation on the rapid-reaction kinetic parameters of the *Pa*DADH E^246^L variant enzyme and whether the mutation resulted in an acquired reactivity of the enzyme with O_2_, the reductive half-reaction of the *Pa*DADH E^246^L variant enzyme was carried out under aerobic conditions. Using an SF-61DX2 Hi-Tech KinetAsyst performance stopped-flow spectrophotometer, the reaction was followed in 20 mM NaPP_i_, pH 10.0, and 25 ^o^C and compared to the anaerobic reductive half-reactions of the wildtype and E^246^L variant enzymes that were previously investigated (42, 70). Since ∼80% of flavin reduction occurs in the mixing time (2.2 ms) of the stopped-flow spectrophotometer with D-arginine as a substrate (42, 71), the flavin reduction of the E^246^L variant enzyme was investigated with D-leucine as the reducing substrate. Substrate solutions (1–40 mM) were loaded into syringes and mounted onto the stopped-flow spectrophotometer. The reaction was followed by observing the spectroscopic decay of the 446 nm flavin peak over time upon reacting ∼10 μM enzyme with substrate solutions under pseudo-first-order conditions in single mixing mode.

## Data analysis

The time-resolved flavin reductions were fit to Equation 1, which describes a triple exponential process for flavin reduction. Here, *k*_obs1_, *k*_obs2_, and *k*_obs3_ represent the observed first-order rate constant for reducing the enzyme-bound flavin at any given substrate concentration at 446 nm. *A* represents the absorbance at 446 nm at any given time, *B*_*1*_, *B*_*2*_, and *B*_*3*_ are the amplitudes of the absorption changes, *t* is time, and *C* is the absorbance at an infinite time that accounts for the nonzero absorbance of the fully reduced enzyme-bound flavin.

(Eq 1) $$A=B_{1}\;\exp\left(-k_{\text{obs}1}t\right)+B_{2}\;\exp\left(-k_{\text{obs}2}t\right)+B_{3}\;\exp\left(-k_{\text{obs}3}t\right)+C$$

The resulting kinetic parameters of the reductive half-reaction were determined after fitting the observed rate constants for flavin reduction at various D-leucine concentrations with Equation 2. The equation defines a hyperbolic saturation of the enzyme with the D-leucine substrate, yielding a *y*-intercept value of zero. The data were fit with the KaleidaGraph software (Synergy Software). Here, *k*_obs1_ represents the observed first-order rate constant for reducing the enzyme-bound flavin at any substrate concentration (*S*). *k*_red_ is the limiting first-order rate constant for flavin reduction at saturating substrate concentrations. *K*_d_ is the apparent equilibrium constant for dissociating the enzyme–substrate complex into the free substrate and enzyme. The same data were obtained when an equation that defines a hyperbolic saturation with a finite *y*-intercept was used.

(Eq 2) $$k_{\text{obs}1}=\frac{k_{\text{red}}S}{K_{d}+S}$$

### O_2_ reactivity studies of the *Pa*DADH E^246^L variant enzyme

To investigate the effect of the E^246^L mutation on the ability of the *Pa*DADH E^246^L variant enzyme to react with O_2_ under steady-state conditions, the initial velocities of the enzyme reaction were measured with D-leucine as a substrate and O_2_ as an electron acceptor at pH 8.5. The reduction of O_2_ was followed using a Clark-type oxygen electrode in a 1 ml reaction volume containing final enzyme concentrations of 0.48 μM to 9.7 μM and fixed D-leucine concentration at 25 mM in 20 mM NaPP_i_ at 25 ^o^C. Substrate solutions were prepared in the reaction buffer, and the pH was readjusted after the amino acid substrate was dissolved. The experiment was repeated with D-arginine as substrate at pH 8.5, in a 1 ml reaction volume containing final enzyme concentrations of 0.17 μM to 8.5 μM and fixed D-arginine concentration at 5 mM in 20 mM NaPP_i_ at 25 ^o^C.

In a separate experiment, the dependence of the *Pa*DADH E^246^L variant enzyme’s O_2_ reactivity on substrate concentration was investigated at fixed 0.5 μM enzyme and varying concentrations of D-arginine (1 mM – 20 mM) or D-leucine (1 mM–10 mM).

To probe the oxygen species generated by the E^246^L variant O_2_ reaction, the enzymatic assay was carried out with 6 μM E^246^L variant enzyme and fixed 5 mM D-arginine or 25 mM D-leucine. The reaction was then repeated by adding 200 to 500 units of superoxide dismutase to the reaction mixture.

To investigate the effect of the *Pa*DADH E^246^L-generated O_2_^•^**ˉ** on the enzyme’s ability to turnover with D-arginine, the steady-state kinetic properties of the variant enzyme with O_2_ as an electron acceptor were investigated using ∼6 μM enzyme with 0.15 mM, 0.24 mM, or 0.5 mM D-arginine. Upon reaching a plateau in the reaction cycle, D-arginine was reintroduced to the reaction mixture to yield a total of four reaction cycles.

### pH effects on the steady-state kinetics of the *Pa*DADH E^246^L variant enzyme

To determine the effects of pH on the steady-state kinetics of the E^246^L variant enzyme of *Pa*DADH, the apparent steady-state kinetic parameters of the enzyme with D-arginine or D-leucine as a substrate and PMS as an artificial electron acceptor were obtained by monitoring the initial PMS-driven O_2_ consumption rates with a computer-interfaced Oxy-32 oxygen-monitoring system (Hansatech Instruments Ltd) under similar conditions. D-Arginine concentrations were between 0.1 and 80 mM, D-leucine concentrations were between 1.25 and 62.5 mM, the enzyme concentration ranged from 1.15 μM to 5.75 μM, PMS concentration was fixed at 1 mM, and the pH ranged from 5.0 to 10.5 at 25 ^o^C. Temperature effects on the steady-state pH profiles for D-arginine were investigated by repeating the assays at 12 ^o^C. All assays were carried out to ensure that the *K*_m_ value was within the range of the substrate concentrations used at each pH. To ensure that the variant enzyme was fully saturated with PMS, the steady-state kinetic parameters were also determined at 1.5 mM PMS, yielding similar results.

Due to the observation of a nonstoichiometric slope of +1.5 for the log (*k*_cat_/*K*_m_) pH profile with D-arginine at 12 ^o^C and 25 ^o^C, the assay was repeated with 200 to 500 units of superoxide dismutase in each apparent steady-state reaction mixture to investigate the effect of the enzyme-O_2_ reactivity on the D-arginine pH profile. The data analyses and interpretation focused only on the *k*_cat_/*K*_m_ pH profiles due to the observation that superoxide dismutase affected only the *k*_cat_/*K*_m_ pH profiles with D-arginine.

For the D-arginine substrate, the log values of the *k*_cat_/*K*_m_ parameters under varying conditions of temperature and superoxide dismutase were plotted by fitting the log values of the *k*_cat_/*K*_m_ parameters with Equation 3. The equation describes a curve that increases with increasing pH with a slope of *S* and a pH-independent limiting value (C_H_) at high pH.

The plot of the *k*_cat_/*K*_m_ parameter for the D-leucine substrate was made by fitting the log values with Equation 4, which describes a curve that increases with increasing pH with a slope of +1 and a pH-independent limiting value (C_H_) at high pH.

(Eq 3) $$\log\;Y=\log\;\frac{C_{H}}{{(1+\frac{10^{-pH}}{10^{{-pK}_{b}}})}^{\wedge }S}$$

(Eq 4) $$\log\;Y=\log\;\frac{C_{H}}{(1+\frac{10^{-pH}}{10^{{-pK}_{b}}})}$$

## Data availability

All data are contained within the manuscript.

## Conflict of interest

The authors declare no conflicts of interest with the contents of this article.

## Footnotes

† Enzymes that react with O_2_ to yield H_2_O_2_ are classified as oxidases while those that react with O_2_ to yield H_2_O are classified as monooxygenases (17, 18). In the case of the *Pa*DADH E^246^L variant enzyme, neither H_2_O_2_ nor H_2_O is produced during turnover with O_2_ as an electron acceptor, instead, O_2_^•^**ˉ** is produced. Hence, the enzymatic activity of the *Pa*DADH E^246^L variant enzyme with O_2_ yielding O_2_^•^**ˉ** has been labeled as an O_2_-driven dehydrogenase activity.
